# Sex Differences in Neck Muscle Mechanical Responses to Writing at a Fixed-Height University Desk

**DOI:** 10.3390/muscles5040077

**Published:** 2026-10-20

**Authors:** Andrea Ježková, Zuzana Hudáková, Jaromír Doležal, Lenka Lhotská, Lucie Loukotová, Hana Kynštová

**Affiliations:** 1 Department of Natural Sciences, Faculty of Biomedical Engineering, Czech Technical University in Prague, nám Sítná 3105, 272 01 Kladno, Czech Republic; 2 Department of Physiotherapy, Faculty of Health Studies, Jan Evangelista Purkyně University in Ústí nad Labem, Sociální Péče 3652/13, 400 96 Usti nad Labem, Czech Republic; 3 Department of Physiotherapy, Faculty of Health, Catholic University in Ružomberok, A. Hlinka Square 48, 034 01 Ruzomberok, Slovakia; 4 Czech Institute of Informatics, Robotics and Cybernetics, Czech Technical University in Prague, Jugoslávských Partyzánů 1580/3, Dejvice, 160 00 Praha, Czech Republic; 5 Department of Mathematics, Faculty of Science, Jan Evangelista Purkyně University in Ústí nad Labem, Pasteurova 3632/15, 400 96 Usti nad Labem, Czech Republic; 6 Department of Occupational Therapy, Faculty of Health Studies, Jan Evangelista Purkyně University in Ústí nad Labem, Sociální Péče 3652/13, 400 96 Usti nad Labem, Czech Republic

**Keywords:** ergonomics, gender differences, muscle stiffness, musculoskeletal disorders, MyotonPRO device, neck pain, static load, women’s health

## Abstract

Introduction Musculoskeletal disorders associated with muscular strain on the neck and shoulders affect women in administration more often than men. Ergonomic guidelines have historically been derived from male models, often ignoring biomechanical differences between the sexes. This study aims to quantify the acute mechanical response of the neck muscles of men and women to a standardized writing task at university desks of the same height and 0° inclination. Methods: The research involved 57 healthy university students (34 women, 23 men; age 19–24 years). Muscle tone (F), stiffness (S), and viscoelastic decrement (D) in the sternocleidomastoideus muscle (SCM), trapezius muscle (UT), and semispinalis capitis muscle (SSC) were assessed by the non-invasive MyotonPRO device before and after 20 min of continuous writing. Results: In the entire cohort, a significant change before and after exercise was found only in the UT decrement (*p* = 0.021). Gender differences were found in the change in muscle tone (ΔF), SCM (*p* = 0.031), and SSC (*p* = 0.039). On average, SCM tone increased slightly in men (+0.120 Hz) and decreased in women (−0.294 Hz), while SSC tone decreased in men (−0.374 Hz) and increased in women (+0.450 Hz). Conclusions: A twenty-minute writing task at a university desk with a fixed height was associated with measurable acute changes in selected mechanical properties of the neck muscles and with differences between the sexes in the direction of change in the tone of SCM and SSC. The clinical relevance of these findings remains unclear and should be validated in larger studies with detailed anthropometric and ergonomic evaluation and longer follow-up.

## 1. Introduction

The current school and work-administrative environment is often designed to be ergonomically universal, with the assumption that the same working conditions will lead to a comparable physiological load for all users [1]. However, this approach implicitly ignores the biological and anthropometric differences between women and men, which can significantly affect muscle strategy, postural control, and response to static workload [2]. As a result, working conditions that are considered neutral may in fact be associated with different demands on the musculoskeletal system. Historically, ergonomic norms for sedentary work have been derived predominantly from male models of body constitution [3]. Although women now make up a substantial part of the workforce in the administrative sector [4], as well as in school institutions, their specific biomechanical characteristics are not systematically taken into account in the design of workplaces. A study with 416 participants showed that women in office work have a significantly higher risk of developing cervical spine pain than men in the same work environment [5]. This difference is unlikely to reflect only one mechanism; workplace dimensions, posture, task length, individual anthropometry, and gender-related physiological characteristics may contribute. Epidemiological evidence suggests that women exhibit different patterns of muscle activation in comparable tasks and are significantly more prone to neck and shoulder girdle overload, leading to a higher prevalence of musculoskeletal disorders [6,7,8]. A possible explanation is that women have different motor movement control strategies and a different response to fatigue, and this may be the cause of a higher incidence of musculoskeletal problems during repetitive work tasks or static postures [9,10]. Ergonomic studies have repeatedly shown that lower imaging/writing planes and flat horizontal plates increase head flexion and alter the activity of the neck and shoulder muscles, especially the upper trapezius and neck extensors. This is already evident in normal office tasks and also when reading/writing on paper or a tablet [11,12,13].

Previous research has relied mainly on subjective questionnaires or surface electromyography (EMG) [7,8,9]. The MyotonPRO device allows an alternative objective quantification of the physical properties of tissue (tone, stiffness, viscoelastic decrement), which are critical precursors of fatigue and pain, while EMG measures current electrical activity. Previous studies using MyotonPRO device technology have confirmed the existence of basal differences in muscle structure between the sexes [14,15,16]; so far, there is a lack of data on how these predispositions determine the immediate biomechanical response to real workload. Our study complements this deficit and provides a perspective on gender-specific management of muscle tension. Therefore, the aim of this preliminary study was to assess changes in the mechanical properties of selected neck muscles before and after a standardized 20-min writing task at a university desk with a fixed height and to verify whether the magnitude and direction of these changes differ between male and female students.

## 2. Results

### 2.1. Common Features of the Mechanical Response

Across the entire cohort, the largest change was observed in the viscoelastic decrement (D) of the trapezius muscle following the 20 min static workload (Table 1). The change was statistically significant (*p* = 0.020), with an estimated change of 0.02 (95% CI: 0.01 to 0.04) and an effect size of 0.32. This observation suggests a change in the muscle’s viscoelastic response after sustained static work in head flexion. Tone (F) and stiffness (S) in the trapezius muscle, and the measured parameters in the other muscles, showed no statistically significant pre–post changes (see Table 1).

### 2.2. Sex Differences

**Figure 1:**
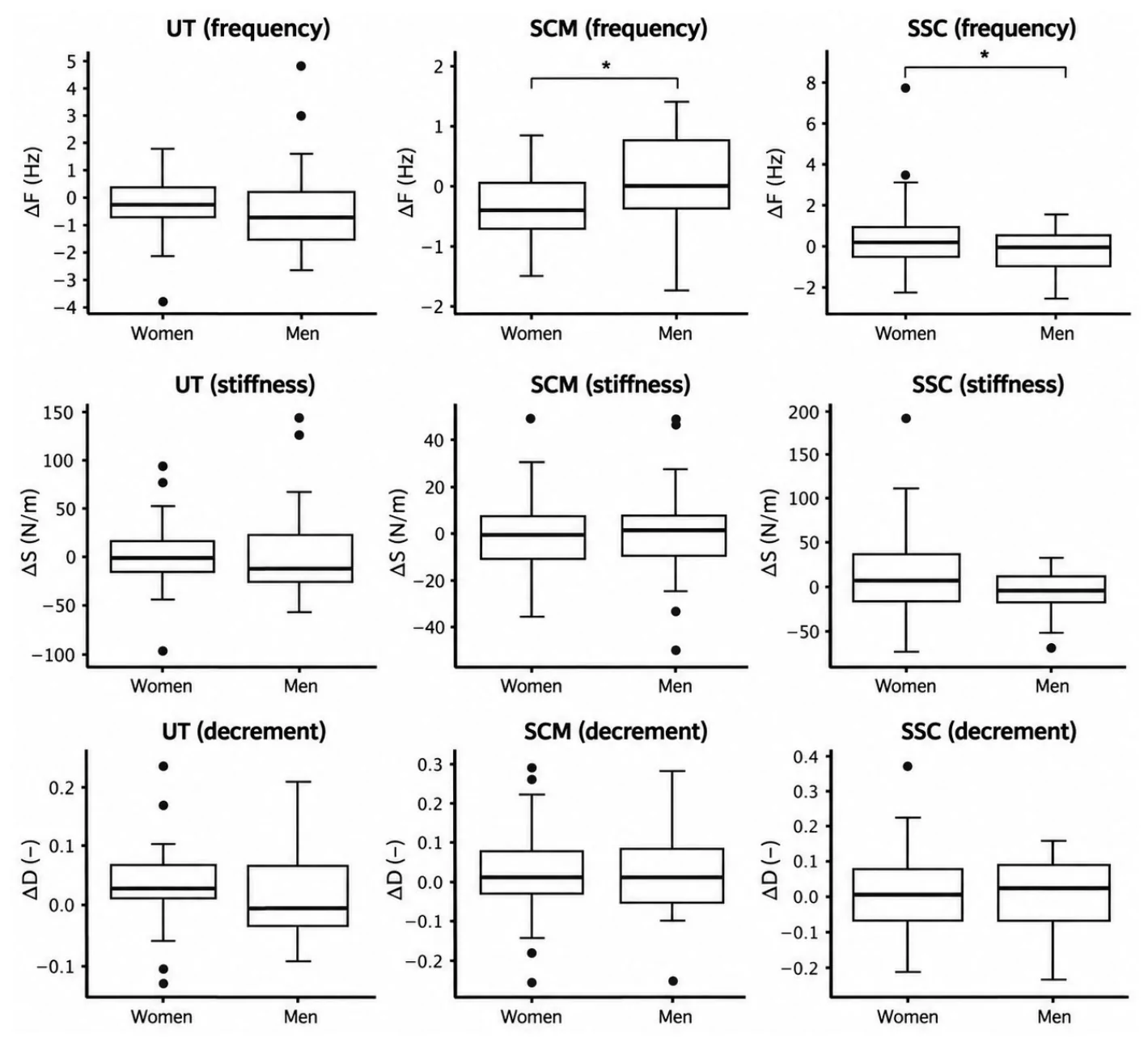
Comparison of muscle mechanical properties (Δ*F*, Δ*S*, Δ*D*) between women and men for m. trapezius (UT), m. sternocleidomastoideus (SCM), and m. semispinalis capitis (SSC). The boxes represent the interquartile range (IQR; 25th–75th percentiles), whiskers extend to 1.5 × IQR, Dots represent outliers (individual observations lying more than 1.5 times the interquartile range beyond the first or third quartile). and horizontal lines indicate the median. * indicates *p* < 0.05.

Although no statistically significant changes were demonstrated in the sternocleidomastoid (SCM) and semispinalis capitis (SSC) muscles in the entire cohort, the sex-specific analysis showed opposite patterns of response (Figure 1, Table 2). For SCM muscle tone, men showed an average increase of 0.120 Hz after the workload, whereas women showed an average decrease of 0.294 Hz. The between-sex difference in mean change was statistically significant (*p* = 0.031; mean difference −0.41 Hz, 95% CI: −0.79 to −0.04 Hz; Cohen’s d = −0.64).

An opposite pattern was observed for SSC muscle tone: women showed an average increase of 0.450 Hz, whereas men showed an average decrease of 0.374 Hz. The between-sex difference in mean change was also statistically significant (*p* = 0.039; mean difference 0.82 Hz, 95% CI: 0.04 to 1.60 Hz; Cohen’s d = 0.52). These findings indicate potentially distinct sex-specific response patterns in the two muscles and provide a basis for further investigation.

### 2.3. The Relationship Between BMI and Parameters

As an additional exploratory analysis, BMI showed a weak positive correlation with the change in sternocleidomastoid muscle tone (ΔF; r = 0.28, *p* = 0.038). No other statistically significant associations between BMI and the measured parameters were reported. This observation suggests a possible relationship between BMI and the response of SCM muscle tone to static loading, warranting further investigation.

### 2.4. Interpretation and Limitations

The observed opposite changes in SCM and SSC muscle tone between women and men suggest different mechanical response patterns to sustained static workload. The corresponding effect sizes and confidence intervals provide information about the magnitude and precision of these findings. However, considerable interindividual variability was observed within both sexes, indicating that these findings represent group-level response patterns rather than uniform responses within each sex. The study included 57 eligible participants, and no a priori sample-size calculation was performed; the sample size may therefore have limited the ability to detect smaller effects. Further studies in larger cohorts are needed to evaluate the reproducibility and physiological relevance of the observed patterns.

## 3. Discussion

The results of our study show that even a short-term writing load lasting 20 min induces measurable changes in the mechanical properties of the neck muscles in students, with the key finding being that while some manifestations of fatigue are common to the whole group, muscle control strategies differ between men and women.

### 3.1. Common Features of the Mechanical Response

The common denominator for all subjects was a significant increase in the decrement parameter in the trapezius muscle, suggesting an increase in tissue viscoelasticity and a reduced ability of the muscle to efficiently accumulate and release mechanical energy. This change is considered an early objective marker of incipient muscle fatigue and deterioration of blood microcirculation [17], which corresponds to earlier studies using EMG, which showed an increase in trapezius activity within 10–20 min of computer work [18]. The fact that these manifestations occur in both sexes in such a short time confirms the significant postural demands even in ordinary administrative tasks.

### 3.2. Sex Differences

While the response of the trapezius muscle was relatively uniform, differences were observed in other neck stabilizers, suggesting distinct responses to static loading between women and men. The mean change in SCM tone was positive in men and negative in women, whereas the opposite pattern was observed for SSC. These findings indicate sex-specific differences in the mechanical response of the cervical muscles to sustained static loading. One possible explanation for these distinct response patterns may involve sex-related differences in anthropometry, muscle characteristics, or motor control. Previous studies have reported differences between women and men in head and neck anthropometry and neck muscle strength [19]. which may influence how static loads are distributed across individual structures of the cervical region. These differences may also be relevant in the context of the higher prevalence of chronic neck pain reported among women working in administrative occupations [4,19,20].

### 3.3. The Relationship Between BMI and Parameters

A supplementary finding of this study is a statistically significant positive correlation (r = 0.281) between BMI and an increase in muscle tone in the sternocleidomastoideus muscle (SCM). The sternocleidomastoideus muscle, as a key muscle of the front of the neck, plays a significant role in stabilizing the head and cervical spine. This positive correlation suggests that individuals with a higher BMI require a greater degree of muscle activation to maintain a static postural position during writing and generate higher muscle tension. This phenomenon may be due to different biomechanics and a higher absolute load on the stabilizing muscles of the neck, or a more frequent occurrence of forward head posture in people with higher body weight. This finding underscores the thesis that universal ergonomic arrangement at the university is insufficient and the physiological response is influenced not only by gender, but also by body constitution.

### 3.4. Implications for School Ergonomics and Future Direction

Based on the measurements made, it can be assumed that repetitive or long-term work of the same nature will have an even worse negative impact on the muscles. It would be appropriate for ergonomically adjustable furniture to also be found in universities and allow for safe adjustment. Our findings correspond to ergonomic studies that recommend individual adjustment of the height of the work surface and a greater inclination of the desk in women to reduce the head flexion angle and thus activate the neck extensors [19,20,21]. In this context, the MyotonPRO device has proven to be a possible reliable and sensitive indicator of changes in muscle properties, which is clearly interpretable for the needs of research and clinical practice, following the example of other studies [22]. To support our results, further steps of the research will lead to a comparison with another objective method and using individually adjusted school furniture.

## 4. Materials and Methods

In this study, the mechanical response of three muscles with different postural functions was monitored. The superficial flexor of the neck muscle sternocleidomastoideus (SCM) (upper third of the muscle belly) was chosen as the primary indicator of the positional adjustment of the head to the torso. M. semispinalis capitis (SSC), (1 cm dorsally from the posterior margin of the SCM, below the level of the processus mastoideus) as a deep extensor, represented a key stabilizing element necessary for controlling the position of the head against gravity in both isometric and reflex tasks [23]. The third monitored muscle was the trapezius muscle (UT) (pars descendens), (the middle of the muscle belly in the triangle between the acromion and the C7 process) as the main postural muscle of the shoulder girdle, whose activity is highly sensitive to workplace ergonomics and which is the primary source of overload during administrative work [21]. The measurement took place exclusively on the dominant side of the body. This approach reflects the natural asymmetry of workload when operating a pen, a mouse, and a keyboard. Monitoring the dominant side thus not only minimizes methodological variability, but also demonstrably better captures the actual level of muscle tension [24].

The duration of the static load was set at 20 min based on current ergonomic findings. Literature sources confirm that measurable changes in posture and muscle activity occur within 10 to 20 min. In addition, continuous work on a horizontal surface beyond this limit leads to excessive cervical flexion and extreme load on the head extensors, which is why it is not recommended in modern ergonomics [11].

### 4.1. Inclusion Criteria

The study included 57 university students (34 women, 23 men; age 19–24) at bachelor’s and master’s levels. All were free of orthopedic or neurological diseases and had no history of significant pain or injuries to the cervical spine in the last 6 months. The body mass index (BMI) of the participants ranged from 18.5 to 30 kg/m^2^. The mean body weight was 58.7 kg (median 57.0 kg; range 44–78 kg) in women and 81.3 kg (median 83.0 kg; range 63.5–100 kg) in men. The mean height was 165.9 cm (median 166.5 cm; range 153–178 cm) in women and 182.2 cm (median 180.0 cm; range 169–198 cm) in men. 42 of the participants were right-handed. Sports activity was reported as moderate; the subjective level of neck discomfort/pain was assessed by the visual analogue pain scale (VAS) before the experiment as 0–2. The measurements took place in the morning, in a standard university environment, at normal room temperature. Each participant signed an informed consent form; the research was approved by the Ethics Committee of Catholic University in Ružomberok, SK, and was conducted in accordance with the Helsinki Declaration. Data collection took place from November 2025 to December 2025.

### 4.2. Measurement Protocol

The measurement was carried out using the MyotonPRO device (Myoton AS, Tallinn, Estonia), which evaluates the mechanical properties of the muscle using a short mechanical pulse (0.4 N, duration 15 ms, frequency 1 Hz). It is a non-invasive, fast, handheld device [25,26]. Each muscle was measured five times; the result was evaluated as a median of five measurements, with a coefficient of variability (CV) of <2% for all muscles, indicating high reliability.

The following parameters were recorded: F (frequency)—muscle tone [Hz], S (stiffness)—muscle stiffness [N/m], D (decrement)—logarithmic decrement, viscoelasticity indicator, any unit.

Experimental procedure: The initial measurement took place in an upright sitting position, with 90° flexion in the hip and knee joints, the upper limbs resting freely with the palms on the thighs, and the soles of the feet fully resting on the ground. Participants looked at the object at eye level. The measured points were marked with markers directly on the skin. The device was placed on the markers with a probe (diameter 3 mm) perpendicular to the muscle belly with constant preload (0.18 N) in order to pre-compress the soft tissues. A rapid, short impulse induced damped tissue oscillations. The device then calculates the parameters defining the properties of the tissue (F, S, D) [17,25]. Muscle tone (F), or muscle tension, is calculated as the maximum frequency; the higher it is, the higher the muscle tension, which increases with contraction or stretching [17,27]. Stiffness (S) is the ability of a muscle to resist an external force; the higher it is, the greater the muscle is able to withstand a greater strain force [17]. A viscoelastic decrement (D) is an indicator of a muscle’s ability to restore its shape and properties after deformation; the smaller it is, the smaller the subsequent dispersion and the more elastic the muscle is [17]. Subsequently, the participant underwent a 20 min continuous load by writing a continuous text on the horizontal board (0° inclination) of the desk. When writing, the upper limbs were resting with their forearms on the table. The height of the desk and chairs was the same for all participants, as well as the situation that occurs in the real environment of universities, where the variability of furniture in lecture halls is not standard. The desk height was 75 cm, while the chair had a seat height of 43 cm, a seat width of 52 cm, and a seat depth of 45 cm. Head posture was monitored by the evaluator visually from the side, specifically the craniovertebral angle (CVA) [28], which is formed by a horizontal line at the level of C7 and a line between C7 and the ear tragus. The participants were instructed to keep the size of the head flexion the same throughout the test. Also, the constant position of the head was verified in the event of an excessive change in the position of the head with a ruler so that the constant distance between the eyes and the plate was about 30 cm. Immediately after the end of writing, the participant straightened up again to the starting position, and repeated measurements of all three muscles were performed at the already marked points.

### 4.3. Statistical Analysis

Basic descriptive statistics (mean, median, minimum, maximum, standard deviation, and interquartile range) were calculated for all monitored variables. Distributional assumptions were assessed using the Shapiro–Wilk test and visually inspected using Q–Q plots. When the assumption of normality was violated, non-parametric methods were used.

Changes between pre-load and post-load measurements for the entire cohort were assessed using a paired *t*-test or, for non-normally distributed differences, the Wilcoxon signed-rank test. For paired *t*-tests, changes are reported as mean paired differences with 95% confidence intervals and effect sizes as Cohen’s d. For Wilcoxon signed-rank tests, Hodges–Lehmann estimates with 95% confidence intervals and rank-biserial correlations are reported.

Sex-specific responses to the workload were assessed using individual change scores calculated as post-load minus pre-load values (Δ = post-load − pre-load). With two measurement occasions, between-sex comparisons of mean change scores directly test the time × sex interaction. Differences in change scores between women and men were assessed using Welch’s independent-samples *t*-test when appropriate. For non-normally distributed change scores, the Mann–Whitney U-test was used to compare change-score distributions. For Welch’s *t*-tests, between-sex differences are reported as differences in mean change with 95% confidence intervals and effect sizes as Cohen’s d. For Mann–Whitney U-tests, Hodges–Lehmann estimates with 95% confidence intervals and rank-biserial correlations are reported.

The analyses were exploratory and aimed to characterize changes in muscle mechanical properties and potential sex-specific response patterns. Results are presented using *p*-values, effect sizes, and 95% confidence intervals to describe the statistical evidence, magnitude, and precision of observed effects. The significance threshold was set at *p* < 0.05, and the findings were interpreted in the context of their exploratory objectives.

All 57 eligible participants available during the data collection period were included. No a priori sample-size calculation was performed; the sample size was determined by participant availability rather than a predefined power requirement. This may have limited the ability to detect smaller effects, particularly time × sex interactions.

Statistical analyses were performed in R (version 4.4.0), and the nominal significance threshold was α = 0.05.

## 5. Conclusions

This study demonstrates that a 20 min writing task performed at a fixed-height university desk with a 0° desktop inclination leads to measurable acute changes in selected mechanical properties of the neck muscles. A statistically significant change in the logarithmic decrement of the trapezius muscle was observed across the entire cohort, while the mechanical responses of the SCM and SSC differed significantly between women and men. These findings suggest that even a relatively short period of continuous writing may affect the mechanical properties of the cervical muscles and that this response may differ between sexes. The results support the importance of considering sex-specific differences and individual ergonomic characteristics when evaluating the effects of prolonged static tasks in academic settings. Further studies with larger samples, detailed ergonomic assessments, and longer or repeated exposures are needed to determine the clinical significance and longer-term implications of these findings.

## Figures and Tables

**Table 1:** Changes in mechanical parameters of neck muscles before and after 20 min static load for the whole group (*n* = 57).

Muscle	Parameter	Before *m* (*SD*)	After *m* (*SD*)	Change Estimate (95% CI)	Effect Size	*p*-Value
UT	*F*	16.67 (1.61)	16.50 (1.91)	0.20 (−0.10, 0.50) ^+^	0.23	n. s.
UT	*S*	285.16 (50.36)	286.88 (63.50)	3.00 (−6.50,11.50) ^+^	0.10	n. s.
UT	*D*	0.88 (0.13)	0.91 (0.15)	0.02 (0.01, 0.04)	0.32	0.020
SCM	*F*	13.03 (0.86)	12.86 (0.90)	−0.17 (−0.35, 0.01)	−0.24	n. s.
SCM	*S*	200.19 (26.06)	199.86 (27.33)	0.33 (−4.93, 5.60)	0.02	n. s.
SCM	*D*	1.21 (0.18)	1.23 (0.16)	0.02 (−0.01, 0.05)	0.15	n. s.
SSC	*F*	14.97 (1.54)	15.09 (2.17)	−0.05 (−0.35, 0.30) ^+^	−0.02	n. s.
SSC	*S*	253.04 (37.28)	257.51 (55.66)	−0.50 (−8.00, 8.00) ^+^	−0.02	n. s.
SSC	*D*	1.25 (0.20)	1.25 (0.19)	0.01 (−0.02, 0.04)	0.07	n. s.

Note: UT = m. trapezius (pars descendens); SCM = m. sternocleidomastoideus; SSC = m. semispinalis capitis; *m* = mean; SD = standard deviation. ^+^ Analyzed using the Wilcoxon signed-rank test; change estimates are Hodges–Lehmann estimates with 95% confidence intervals and effect sizes are rank-biserial correlations. For all other variables, paired *t*-tests were used; change estimates are mean paired differences with 95% confidence intervals and effect sizes are Cohen’s d. Reported *p*-values are unadjusted; n. s. indicates *p* > 0.05.

**Table 2:** Between-sex differences in changes in mechanical parameters of neck muscles following 20 min static load (*n* = 57).

Muscle	Parameter	Δ Women *m* (*SD*)	Δ Men *m* (*SD*)	Between-Sex Difference in Change (95% CI)	Effect Size	*p*-Value
UT	*F*	−0.16 (1.09)	−0.20 (1.68)	0.30 (−0.4, 0.9) ^+^	0.15	n. s.
UT	*S*	0.62 (35.21)	3.35 (50.33)	6.50 (−13.00, 23.00) ^+^	0.09	n. s.
UT	*D*	0.03 (0.07)	0.01 (0.08)	0.03 (−0.02, 0.06) ^+^	0.16	n. s.
SCM	*F*	−0.29 (0.58)	0.12 (0.75)	−0.41 (−0.79, −0.04)	−0.64	0.031
SCM	*S*	−0.79 (18.29)	0.35 (22.35)	−1.14 (−12.49, 10.20)	−0.06	n. s.
SCM	*D*	0.02 (0.11)	0.02 (0.12)	0.03 (−0.06, 0.07)	0.03	n. s.
SSC	*F*	0.45 (1.8)	−0.37 (1.10)	0.82 (0.04, 1.60)	0.52	0.039
SSC	*S*	12.97 (49.94)	−8.01 (22.6)	12.00 (−3.00, 31.00) ^+^	0.26	n. s.
SSC	*D*	0.01 (0.13)	0.01 (0.11)	0.001 (−0.06, 0.06)	0.01	n. s.

Note: UT = m. trapezius (pars descendens); SCM = m. sternocleidomastoideus; SSC = m. semispinalis capitis; *m* = mean; SD = standard deviation; Δ = post-load − pre-load change. ^+^ Analyzed using the Mann–Whitney U-test; between-sex differences in change are reported as Hodges–Lehmann estimates with 95% confidence intervals and effect sizes as rank-biserial correlations. For all other variables, Welch’s independent-samples *t*-tests were used; between-sex differences in change are reported as differences in mean change with 95% confidence intervals and effect sizes as Cohen’s d. Reported *p*-values are unadjusted; n. s. indicates *p* > 0.05.

## Data Availability

The data presented in this study are available from the corresponding author upon reasonable request.

## References

[1] Aiyegbusi A.I., Gbiri C.A., Oyeniran T.O., Balogun O.J. (2023). Mismatch between School Furniture Dimensions and Anthropometric Parameters Is a Risk for Spinal Deformities in Secondary School Students in Lagos, Nigeria: A Cross-Sectional Study. Bull. Fac. Phys. Ther..

[2] Eby S.F., Cloud B.A., Brandenburg J.E., Giambini H., Song P., Chen S., LeBrasseur N.K., An K.-N. (2015). Shear Wave Elastography of Passive Skeletal Muscle Stiffness: Influences of Sex and Age throughout Adulthood. Clin. Biomech..

[3] Criado-Perez C. (2019). Invisible Women: Data Bias in a World Designed for Men.

[4] Eurostat Eurostat 4 March 2024. Jobs with the Highest Shares of Women in Q3 2023 (Statistic Data). https://ec.europa.eu/eurostat/web/products-eurostat-news/w/ddn-20240304-1.

[5] Korhonen T., Ketola R., Toivonen R., Luukkonen R., Häkkänen M., Viikari-Juntura E. (2003). Work Related and Individual Predictors for Incident Neck Pain among Office Employees Working with Video Display Units. Occup. Environ. Med..

[6] Anders C., Bretschneider S., Bernsdorf A., Erler K., Schneider W. (2004). Activation of Shoulder Muscles in Healthy Men and Women under Isometric Conditions. J. Electromyogr. Kinesiol..

[7] Fedorowich L., Emery K., Gervasi B., Côté J.N. (2013). Gender Differences in Neck/Shoulder Muscular Patterns in Response to Repetitive Motion Induced Fatigue. J. Electromyogr. Kinesiol..

[8] Shin E., Roh H. (2024). Gender-Based Analysis of Muscle Activation While in a Sedentary Work Posture. Technol. Health Care.

[9] Srinivasan D., Sinden K.E., Mathiassen S.E., Côté J.N. (2016). Gender Differences in Fatigability and Muscle Activity Responses to a Short-Cycle Repetitive Task. Eur. J. Appl. Physiol..

[10] Weber Z.R., Srinivasan D., Côté J.N. (2018). Sex-Specific Links in Motor and Sensory Adaptations to Repetitive Motion–Induced Fatigue. Mot. Control.

[11] Bhuanantanondh P., Rungkitlertsakul S., Richards J. (2025). Is Neck and Shoulder Posture, Muscle Activity and Discomfort Influenced by Tablet Inclination in Young Adults with and without Neck Pain?. PLoS ONE.

[12] Intolo P., Shalokhon B., Wongwech G., Wisiasut P., Nanthavanij S., Baxter D.G. (2019). Analysis of Neck and Shoulder Postures, and Muscle Activities Relative to Perceived Pain during Laptop Computer Use at a Low-Height Table, Sofa and Bed. Work.

[13] Farias Zuniga A.M., Côté J.N. (2017). Effects of Dual Monitor Computer Work Versus Laptop Work on Cervical Muscular and Proprioceptive Characteristics of Males and Females. Hum. Factors J. Hum. Factors Ergon. Soc..

[14] Mencel J., Jaskólska A., Marusiak J., Kisiel-Sajewicz K., Siemiatycka M., Kaminski L., Jaskólski A. (2021). Effect of Gender, Muscle Type and Skinfold Thickness on Myometric Parameters in Young People. PeerJ.

[15] Tashiro T., Okugaki T., Abekura T., Kumamoto S., Arima S., Maeda N. (2025). Sex and Side Differences in Muscle Stiffness of Lower Limb Muscles in Healthy Adults. Gait Posture.

[16] Dietsch A.M., Clark H.M., Steiner J.N., Solomon N.P. (2015). Effects of Age, Sex, and Body Position on Orofacial Muscle Tone in Healthy Adults. J. Speech Lang. Hear. Res..

[17] Gavronski G., Veraksitš A., Vasar E., Maaroos J. (2007). Evaluation of Viscoelastic Parameters of the Skeletal Muscles in Junior Triathletes. Physiol. Meas..

[18] Szeto G.P.Y., Straker L.M., O’Sullivan P.B. (2005). A Comparison of Symptomatic and Asymptomatic Office Workers Performing Monotonous Keyboard Work—1: Neck and Shoulder Muscle Recruitment Patterns. Man. Ther..

[19] Vasavada A.N., Danaraj J., Siegmund G.P. (2008). Head and Neck Anthropometry, Vertebral Geometry and Neck Strength in Height-Matched Men and Women. J. Biomech..

[20] Côté J.N. (2012). A Critical Review on Physical Factors and Functional Characteristics That May Explain a Sex/Gender Difference in Work-Related Neck/Shoulder Disorders. Ergonomics.

[21] Straker L., Burgess-Limerick R., Pollock C., Murray K., Netto K., Coleman J., Skoss R. (2008). The Impact of Computer Display Height and Desk Design on 3D Posture during Information Technology Work by Young Adults. J. Electromyogr. Kinesiol..

[22] Volesky K., Novak J., Janek M., Katolicky J., Tufano J.J., Steffl M., Courel-Ibáñez J., Vetrovsky T. (2025). Assessing the Test-Retest Reliability of MyotonPRO for Measuring Achilles Tendon Stiffness. J. Funct. Morphol. Kinesiol..

[23] Siegmund G.P., Blouin J.-S., Brault J.R., Hedenstierna S., Inglis J.T. (2007). Electromyography of Superficial and Deep Neck Muscles During Isometric, Voluntary, and Reflex Contractions. J. Biomech. Eng..

[24] Diederichsen L.P., Nørregaard J., Dyhre-Poulsen P., Winther A., Tufekovic G., Bandholm T., Rasmussen L.R., Krogsgaard M. (2007). The Effect of Handedness on Electromyographic Activity of Human Shoulder Muscles during Movement. J. Electromyogr. Kinesiol..

[25] Agyapong-Badu S., Warner M., Samuel D., Stokes M. (2016). Measurement of Ageing Effects on Muscle Tone and Mechanical Properties of Rectus Femoris and Biceps Brachii in Healthy Males and Females Using a Novel Hand-Held Myometric Device. Arch. Gerontol. Geriatr..

[26] Lee M.-T., Wu C.-Y., Chen C.-W., Cheng H.-L., Chen C.-C., Hsieh Y.-W. (2022). Age and Sex Differences in the Biomechanical and Viscoelastic Properties of Upper Limb Muscles in Middle-Aged and Older Adults: A Pilot Study. J. Biomech..

[27] Ditroilo M., Hunter A.M., Haslam S., De Vito G. (2011). The Effectiveness of Two Novel Techniques in Establishing the Mechanical and Contractile Responses of Biceps Femoris. Physiol. Meas..

[28] Yong M.-S., Lee H.-Y. (2025). Does Forward Head Posture Influence Muscle Tone, Stiffness, and Elasticity in University Students?. J. Clin. Med..

